# Transitioning to Web-Based Learning in Basic Life Support Training During the COVID-19 Pandemic to Battle the Fear of Out-of-Hospital Cardiac Arrest: Presentation of Novel Methods

**DOI:** 10.2196/27108

**Published:** 2021-05-25

**Authors:** Katarzyna Naylor, Kamil Torres

**Affiliations:** 1 Department of Didactics and Medical Simulation Medical University of Lublin Lublin Poland

**Keywords:** COVID-19, web-based training, basic life support, formative assessment, out-of-hospital cardiac arrest, web-based learning, web-based education, first aid, medical education, life support, transition, outcome, formative

## Abstract

Ongoing training in the area of basic life support aims to encourage and sustain the willingness to act in out-of-hospital cardiac arrest situations among first aiders. The contribution of witnesses and first aiders has diminished rapidly, as suspicion associated with the COVID-19 pandemic has risen. In this paper, we present teaching methods from the medical education field to create a new teaching-learning process for sustaining the prehospital involvement of first aiders and encourage new first aiders. The most important benefit—improving outcomes—can be achieved by introducing a variety of teaching-learning methods and formative assessments that provide participants with immediate feedback to help them move forward in the basic life support course. The new reality of web-based learning that has been introduced by the pandemic requires an innovative approach to traditional training that involves techniques and methods that have been proven to be useful in other fields.

## Introduction

Due to public health concerns in the wake of 2020, which were caused by the novel COVID-19, face-to-face contact in medical training was promptly substituted with remote teaching. The teaching-learning process was moved to the houses of participants through a variety of learning management systems and videoconferencing services [1]. Similarly, all hands-on resuscitation training in the form of in-person, hands-on sessions was stopped [2]. Nevertheless, the European Resuscitation Council (ERC) as well as the International Liaison Committee on Resuscitation (ILCOR), in their educational update in April 2020 on teaching during a pandemic, highlighted the importance of sustaining sudden cardiac arrest training in some form, despite the modified conditions for knowledge transfer. The ILCOR and national societies underlined the significance of continuing education to improve resuscitation knowledge, certain skills, and, most importantly, patient-centered care to sustain and encourage the willingness to act during out-of-hospital cardiac arrest (OHCA) situations [3,4]. Complementary basic life support (BLS) teaching methods involving computer-based and video-based e-learning or the gamification approach have been proven to be useful in the area of BLS [5,6]. However, the cost-effectiveness and standardization of the training delivered via these educational methods should also be considered [7,8].

The long-term retention of BLS competencies is essential and outweighs skills performance during teaching sessions, according to published evidence [9,10]. Such pieces of evidence have opened a new door in the era of BLS training. Additionally, as BLS training is conducted in each health care professional undergraduate medical curriculum, there have been additional demands for finalizing modules that were planned for educational months, which changed due to the COVID-19 pandemic. The Polish Ministry of Health, together with the Ministry of Education, suspended face-to-face education from March 12, 2020, onward [11]. Therefore, the opportunity for launching a web-based BLS course arose (e-BLS) in response to new conditions.

To expand people’s knowledge about distant and web-based possibilities for learning in the area of BLS, we present a proposal for a newly designed e-learning course about BLS competencies. This course was developed to (1) enable participants to understand and cultivate the necessary competencies in each of the OHCA domains and (2) promote the implementation of the steps required for helping people in need of BLS. The course design entails a novel teaching methodology that has been extensively researched in the medical education field.

## Methods

### The Goal Of Training and Its Objectives

A multimodal, participant-centered, interactive, web-based course for addressing the unique challenges of sudden cardiac arrest that a layperson may face daily was developed. The overarching goals of the course are to (1) enable participants to understand and cultivate the necessary competencies in each of the OHCA domains and (2) promote the implementation of the steps required for helping people in need of BLS. Specific objectives were determined by analyzing the ERC guidelines update for the COVID-19 pandemic. This was done to set up BLS course learning objectives and the needs assessment of the participants (Table 1).

**Table 1:** Objectives, instructional methods, and implementation strategies for each basic life support (BLS) competency session.

Session topic (number of sessions; time of each session)	Objectives	Instructional design and implementation
Safety (2; 45 minutes)	Personal safety and the safety of people in need of BLS in cardiac arrest situations during the COVID-19 pandemic (hand hygiene, the donning and doffing of gloves and masks, types of masks, methods for approaching a person in need of BLS, and scene safety)	Video-based training [12]: Students review the instructional videos on the e-learning platform, produce instructional videos to present their skills, and receive feedback from the faculty on their performance.
BLS (3; 45 minutes)	Standard algorithms and modifications resulting from pandemic (methods that do not involve the look, listen, and feel technique and continuous, chest compression–only CPR^a^ without mouth-to-mouth ventilation)	Video-based training and decision trees [13]: After familiarizing themselves with the assigned materials and conducting video discussions, students are presented with selected sudden cardiac arrest recognition and management scenarios related to BLS. Students have 5 attempts to solve each of the two decision trees and receive immediate feedback.
AED^b^ (3; 45 minutes)	Description of the equipment, equipment use, and where and how to find equipment during an out-of-hospital cardiac arrest Using an AED as a nonaerosol-producing step	Video-based training and decision trees: After familiarizing themselves with the assigned materials and conducting video discussions, students are presented with selected sudden cardiac arrest recognition and management scenarios related to BLS. Students have 5 attempts to solve decision trees and receive immediate feedback. Simple game scenario [6]: Participants implement the BLS/AED algorithm in a simple virtual environment, and the player or student has to save a person by applying CPR actions. Find your AED: Participants find the closest device to their place of residence by using the Staying Alive app [14].
pBLS^c^ and FBAO^d^ (3; 45 minutes)	Standard algorithms and modifications resulting from the pandemic	Video-based learning and decision trees: After familiarizing themselves with the assigned materials and conducting video discussion, students are presented with selected pediatric sudden cardiac arrest recognition and management scenarios related to pBLS. Students have 5 attempts to solve 1 decision tree and receive immediate feedback. Instructor-led, live practice session on Zoom about FBAO algorithms for infants and chest compressions for infants: Participants use available toys resembling a newborn.
Special circumstances leading to sudden cardiac arrest, such as anaphylaxis, heart attacks, strokes, diabetes, drowning, hypothermia, burns, and seizures (3; 45 minutes)	Definitions, standard algorithms, and modifications resulting from the pandemic	Peer assessments [15] and teacher assessments of the presentations of assigned topics are prepared in a group of 3 people and recorded by participants.
Review (4; 45 minutes)	Review of all of the topics	The Script Concordance Test [16,17]: This includes 8 BLS scenarios accompanied by questions concerning possible next steps (Multimedia Appendix 1). Each of the questions is supplemented by a new piece of information concerning the sudden cardiac arrest health issue being considered. Students use a 3-point Likert scale to determine whether and to what extent the new piece of information influences further evaluations.

^a^CPR: cardiopulmonary resuscitation.

^b^AED: automated external defibrillator.

^c^pBLS: pediatric basic life support.

^d^FBAO: foreign body airway obstruction.

### Participants: Target Group

The course aims to provide an introduction to BLS and strengthen the elements of the chain of survival. No professional BLS experience is required. However, the course can also be treated as a refresher course for maintaining participants’ motivation to assist in OHCA situations and providing an update on BLS training to health professionals. Therefore, the target population includes students of medical faculties (medicine, dentistry, nursing, midwifery, biomedicine, paramedics, public health, and dieticians), first aiders with initial first aid knowledge, lay rescuers who are enrolled in a first aid course, and health care providers who want to refresh their knowledge.

The e-learning modules have been embedded in the curriculum of students in all medical faculties of the local medical university, as first aid is obligatory in all curricula. The decision trees and the Script Concordance Test (SCT) have been prepared in Polish and English language for Polish and foreign students. The course is planned for 20 teaching hours and takes place in an academic environment.

To target other professional groups and voluntary first aiders, a massive, open, web-based course will be established to enable their participation and access to the proposed activities.

### Evaluation

The course will be assessed on the basis of participants’ achievements on the final, summative, multiple-choice question test; the SCT; and decision trees as well as data from an anonymous survey based on the Utrecht Seminar Evaluation questionnaire by Spruijt et el [18]. The authors made the tool available upon our request.

### Learning Management Systems and Videoconferencing Services

**Figure 1:**
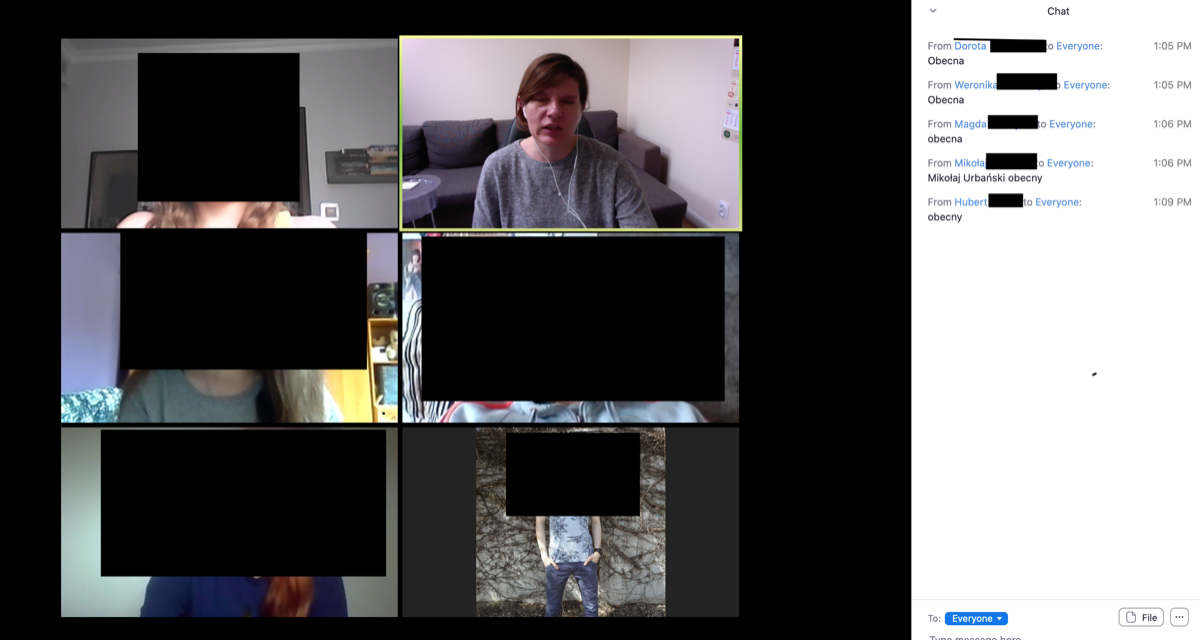
An anonymized screen shot from an individual Zoom meeting.

**Figure 2:**
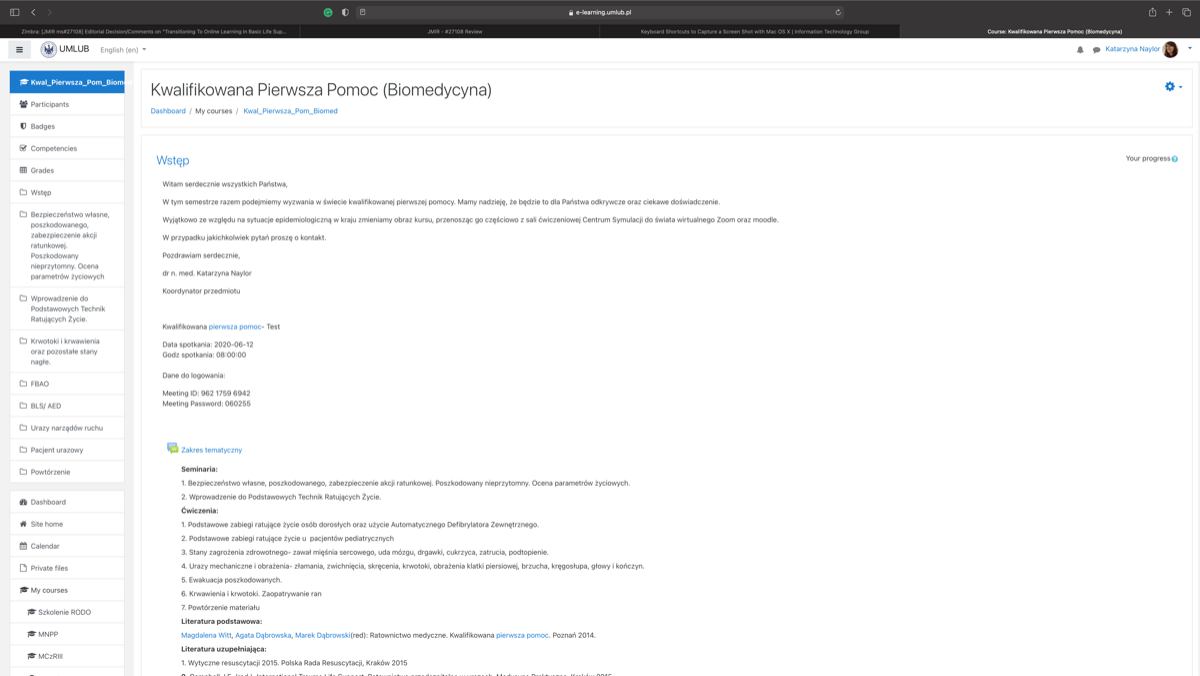
A general overview of an introductory Moodle course website.

**Figure 3:**
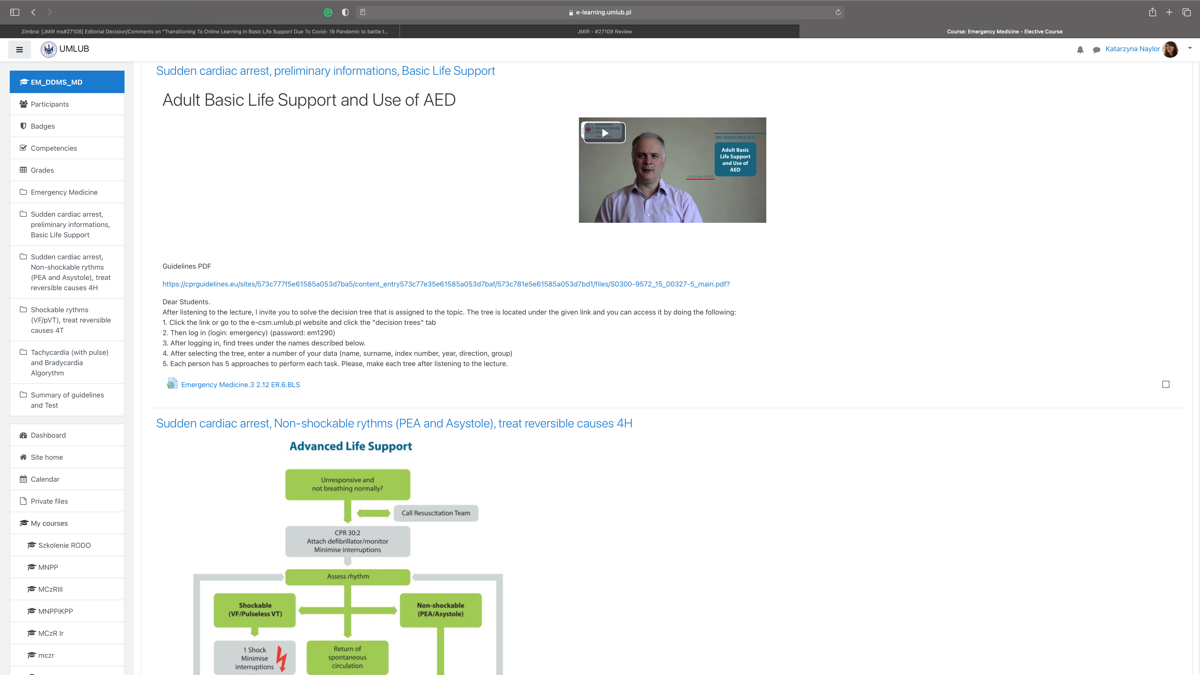
A screenshot of another course on Moodle and a decision tree for instruction. AED: automated external defibrillator; PEA: pulseless electrical activity; VF: ventricular fibrillation; VT: ventricular tachycardia.

Based on the advice of experienced researchers, two platforms that are well known to participants were chosen to host the course—Zoom (Zoom Video Communications Inc; Figure 1) and Moodle (Figures 2 and 3)—to alleviate uncertainty and distrust among the students [19]. Zoom enabled live meetings with participants for discussing a given topic, whereas Moodle, a web-based guiding platform, was used to store all of the information on the course meetings and requirements and the organization, all of the materials and resources assigned to topics, and the links to the formative tasks described in this paper. To adjust to web-based classes, participants received detailed instructions on the use of both platforms. The decision trees’ software constituted another university software; however, links to each task appeared on Moodle and were assigned to a given subject (BLS, BLS/automated external defibrillator [AED], or pediatric BLS), and detailed instructions on how to access the task as well as the rules of engagement were provided.

The educational game for engaging with BLS/AED drills was also located on a different server. A freely available resource was used [20]. Elements of the game were scored, and participants posted their obtained results on the Moodle forum.

**Figure 4:**
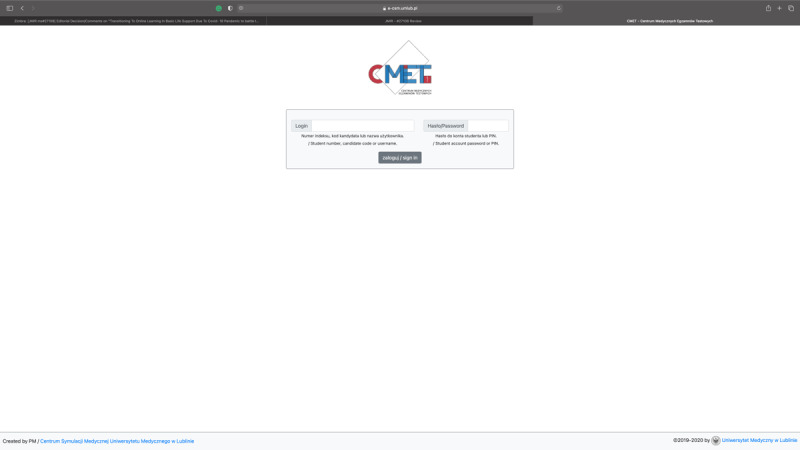
A screenshot of a login page to the CMET website. CMET: Centrum Medycznych Egzaminów Testowych.

**Figure 5:**
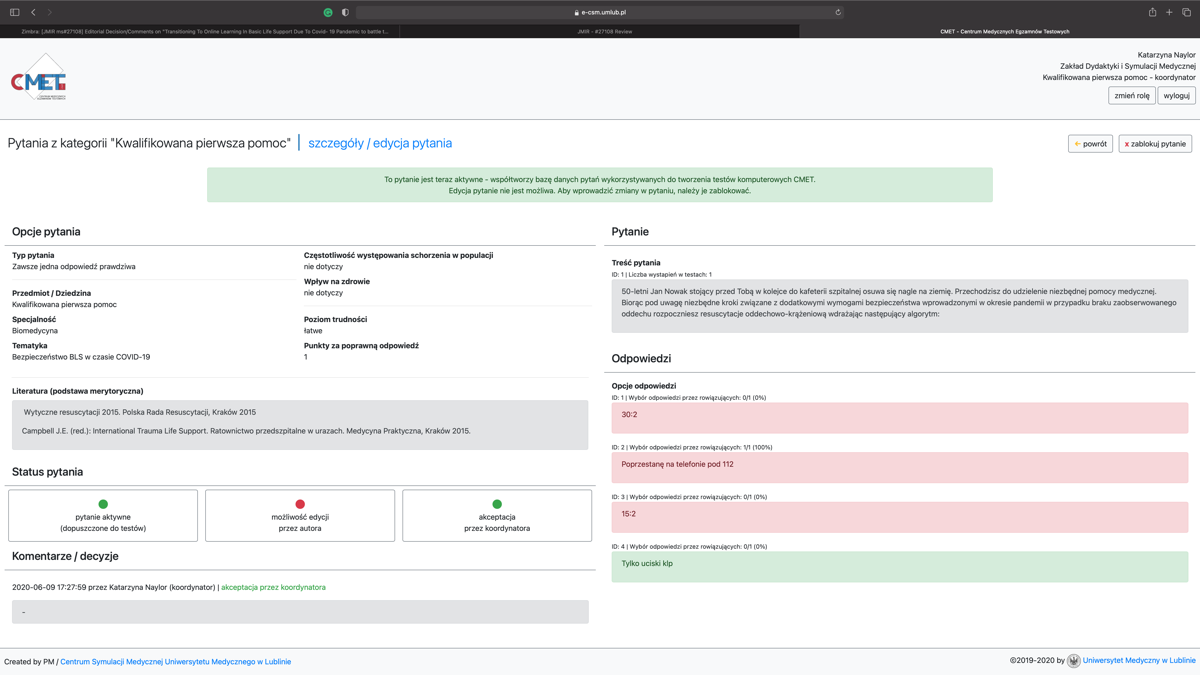
A screenshot of a coordinator account page for a single question on the CMET website. CMET: Centrum Medycznych Egzaminów Testowych.

Another type of software that was used during the course was a newly developed testing system—the Testing Centre for Medical Exams (Centrum Medycznych Egzaminów Testowych [CMET]) [21]. The final summative assignment was held on CMET. The CMET used the following 3-level system for constructing the summative assessment (multiple-choice questions): (1) teachers input the questions into the system; (2) specialists in the area reviewed the questions; and (3) the course coordinator accepted, edited, or sent back the questions (with comments) to their primary creator. This was done to ensure the quality of the provided tasks and generate a more complex task (Figures 4 and 5).

Before the final assignment, the participants receive access to a practice test to confirm their login details and familiarize themselves with the system’s construction. During the test, each student is individually timed during their attempt and receives immediate feedback on the questions alongside their results after they finalize their attempt.

## Discussion

### Content and Facilitation

The implemented assessment points support learning and correspond with the implemented content. Our assessments points focus on formative assessment; they are not used as a tool for passing judgment and making pass-fail decisions but as a tool for assisting with and supporting learning [22]. Formative assessments are also known as assessments for learning or learning-oriented assessments. Such assessments aim to provide feedback on understanding and help participants move forward in the course (Table 1).

### Novel Assessment Formats That Promote Knowledge Acquisition

Learning is promoted through the formative tasks in the outlined BLS web-based course. Participants need to be consciously analyzing and applying gathered “intel” on a given subject in order to benefit from the experience. All of the proposed types of formative assessments in our course aim to produce feedback on performance to advance the learning of participants [23].

The American Heart Association (AHA) has implemented a video-based approach (a teaching approach centered on using videos) that allows AHA course participants to practice BLS techniques while watching videos (ie, practice-while-watching method) during AHA courses. The AHA also provides the option of an e-learning module, in which participants familiarize themselves with the content of videos and textual content before participating in hands-on sessions [24]. Additionally, the ERC has focused on implementing the Peyton approach (ie, a 4-step method) during their hands-on sessions [25]. Therefore, the focus of BLS courses was placed on face-to-face training, and web-based materials were only used to prepare for face-to-face meetings.

### The SCT

The SCT as an assessment method for evaluating clinical reasoning that has several advantages in BLS training. Students tackle a genuine case to demonstrate their ability to incorporate new data into the information on the provided scenario [16,17]. They can compare their reasoning and conduct related discussions, thereby allowing them to learn from each other. This is especially important in emergency situations and OHCA situations, in which quick and timely decisions are required. Additionally, the SCT goes beyond pure fact checking; it requires logical thinking and knowledge application based on procedural knowledge (Multimedia Appendix 1).

### Decision Trees

**Figure 6:**
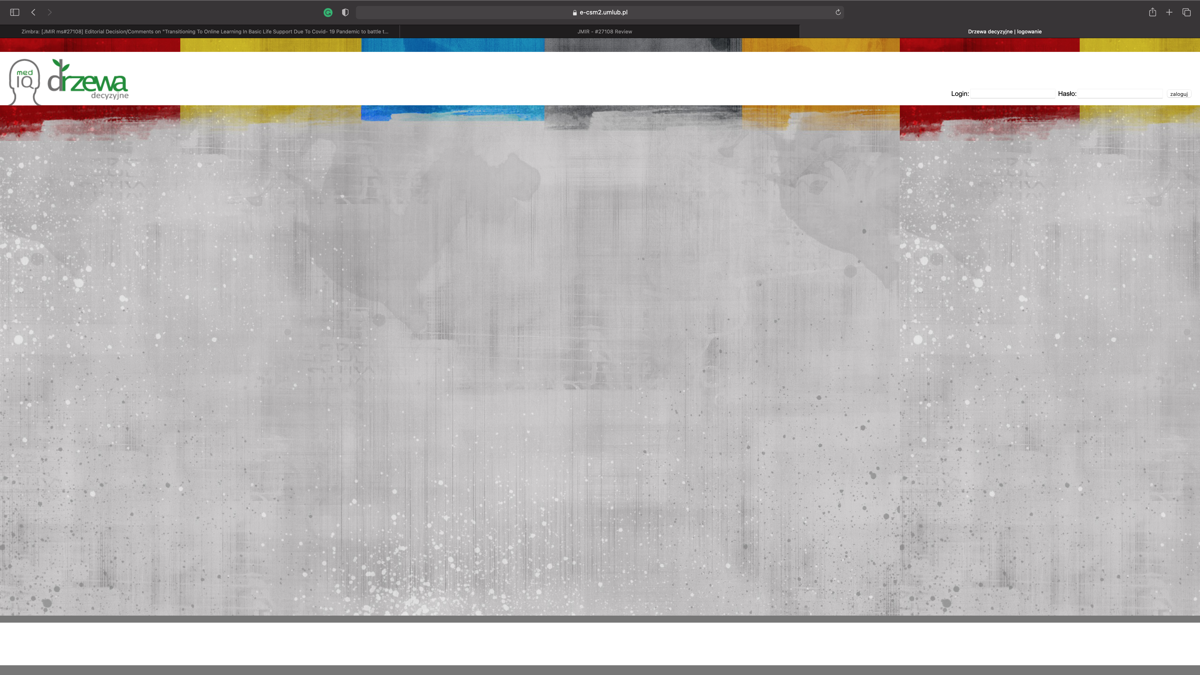
A screenshot of a login website for accessing the decision trees.

Decision trees are based on the assumption that machine learning algorithms are helpful in the medicine field, as errors can have a dire consequence. Decision trees have been used in the decision-making process in clinical settings [13,26]. Nevertheless, the existing system allows first aiders to tackle the complexities and uncertainties of OHCA. Existing decision tress allow for the implementation of knowledge at the more critical level of the Millers pyramid in BLS competency training (Multimedia Appendix 2; Figure 6).

### Gamification

﻿The BLS/AED game was designed to complement BLS teaching and refresh skills in a unique, enjoyable way [6]. The game presents an OHCA scenario by using simple graphics and takes players to a virtual environment where they make choices based on the steps of the BLS/AED algorithm (Multimedia Appendix 2). The game is educational and is a unique medium for knowledge transmission; such games have been proven to promote active learning, help with solving clinical problems, and help learners gain experience in a risk-free environment [6]. At the end of the game, the participants of our course will receive feedback on their performance, and they will have an opportunity to undergo another attempt (Multimedia Appendix 3).

### Conclusions

There is a large variety of web-based first responder and BLS courses. Although the new reality resulting from a pandemic and teaching and learning during a pandemic can have difficulties, they also create new possibilities. Enabling continuous BLS training with appropriate tools, including a variety of didactic methods and assessment formats, can facilitate an uninterrupted process of learning for battling the fear of out-of-hospital resuscitation during the COVID-19 pandemic. However, the modifications in distant BLS learning are at an early stage, and there is a need for robust research that determines (1) their association with participant outcomes; (2) their impact on OHCA; and (3) whether such methodologically diverse learning is cost-effective. Addressing these issues will provide further insight into the role and effectiveness of new technologies and their potential impact on acquiring and sustaining BLS competencies.

### Possible Implications for Practice

First, during the pandemic, the OHCA and BLS curricula require more care for nurturing and supporting competencies in the area of public health. Second, an integrated web-based program requires the combination of modern technology and formative assessments that are dedicated to developing critical thinking and decision-making skills that encourage people to take action in OHCA situations. Third, appropriate modern resources are an integral part of creating modern curricula for BLS that encourage people to take action in OHCA situations.

### Limitations

One of the limitations of our research may be that a lot of time is required to complete all of the formative activities. An increase in the amount of time devoted to a given training course puts an additional burden on course participants. However, well-thought-out activities that mirror real-life situations should encourage participants to spend more time in tackling such activities.

## Appendix

Multimedia Appendix 1 The proposed Script Concordance Test.

Multimedia Appendix 2 A recording of an exemplary decision tree used during the course.

Multimedia Appendix 3 A recording of the game used during the course. Audio commentary in English is provided.

## Abbreviations

AED: automated external defibrillator
AHA: American Heart Association
BLS: basic life support
CMET: Centrum Medycznych Egzaminów Testowych
ERC: European Resuscitation Council
ILCOR: International Liaison Committee on Resuscitation
OHCA: out-of-hospital cardiac arrest
