# Supplementary material for: Transitioning to Web-Based Learning in Basic Life Support Training During the COVID-19 Pandemic to Battle the Fear of Out-of-Hospital Cardiac Arrest: Presentation of Novel Methods
Source: J Med Internet Res. 2021 May 25;23(5):e27108. doi: 10.2196/27108 (PMC8153032; doi:10.2196/27108)
Supplement: Multimedia Appendix 1 [file jmir_v23i5e27108_app1.docx]

**Multimedia Appendix 1.** The proposed Script Concordance Test.

| 50-year-old Jan Nowak, standing in front of you in the queue to the hospital cafeteria, collapses on the ground. You proceed to deliver the necessary medical assistance. | | | | |
| --- | --- | --- | --- | --- |
| **If you were**  **thinking of:** | **And then:** | **This initial decision**  **becomes:** | | |
| Sudden Cardiac Arrest, based on the absence of breath and heart rate. | You notice irregular attempts of catching air  in the victim. | -1  contraindicated | 0  neither more or less indicated | 1  completely indicated |
| Initiating CPR  in a ratio of 30: 2. | A nurse comes running from a nearby ward with a bag-mask device. | -1  contraindicated | 0  neither more or less indicated | 1  completely indicated |
| Ceasing CPR after 4 minutes due to  AED information: defibrillation not recommended. | You notice the spontaneous movements of the victim's chest | -1  contraindicated | 0  neither more or less indicated | 1  completely indicated |

| During a conversation with yourself, 45-year-old Zenon Litwin, your neighbor, suddenly loses consciousness while mowing the loan in his garden. You check consciousness: U on the AVPU scale, then ABC - no signs of life. | | | | |
| --- | --- | --- | --- | --- |
| **If you were**  **thinking of:** | **And then:** | **This initial decision**  **becomes:** | | |
| Sudden Cardiac Arrest, implementing Hands-Only- CPR and asking your colleague present at the scene for help. | The neighbor's wife informs you that he had experienced an episode of a heart attack in the past. | -1  contraindicated | 0  neither more or less indicated | 1  completely indicated |
| Continuing Hands-Only- CPR. | A colleague delivers an AED from a nearby Fire Station. | -1  contraindicated | 0  neither more or less indicated | 1  completely indicated |
| After a shock to continue Hands-Only- CPR due to absent ABC. | After the next cycle of CPR, AED provides information: “defibrillation not recommended." | -1  contraindicated | 0  neither more or less indicated | 1  completely indicated |

| In the laboratory where you work, one of your coworkers, Neil Smith, has suddenly been bitten by an insect. He suffers from shortness of breath, and he asks you for urgent help. | | | | |
| --- | --- | --- | --- | --- |
| **If you were**  **thinking of:** | **And then:** | **This initial decision**  **becomes:** | | |
| Anaphylactic shock. | The coworker informs you he is allergic to paracetamol. | -1  contraindicated | 0  neither more or less indicated | 1  completely indicated |
| Taking the coworker to the closest ER while continuing the SAMPLE interview while. | Suddenly he loses consciousness. | -1  contraindicated | 0  neither more or less indicated | 1  completely indicated |
| Sudden Cardiac Arrest, implementing Hands-Only- CPR. | You notice spontaneous chest movements. | -1  contraindicated | 0  neither more or less indicated | 1  completely indicated |
| A 24-year-old Jack Nuck, your colleague, visited you to return the book he borrowed. However, he starts to look pale, he complains about malaise, facial swelling, cold sweats. Jack admits that he underwent the procedure of removal (extraction) of his wisdom tooth about an hour ago. | | | | |
| **If you were**  **thinking of:** | **And then:** | **This initial decision**  **becomes:** | | |
| Continuing SAMPLE interview to investigate whether it is not anaphylaxis. | Jack informs you  that he took 1 tablet of Ketonal (a medicine he has used several times before) | -1  contraindicated | 0  neither more or less indicated | 1  completely indicated |
| After completing the interview, you suggest giving your friend a lift to the dentist’s office where the procedure was performed to exclude any complications. | In the meantime, you notice a skin rash around the extracted tooth, Jack starts to have slurry speech, feel sick and dizzy. | -1  contraindicated | 0  neither more or less indicated | 1  completely indicated |
| You assist your college in your car to take him to the closest ER. | During the drive, Jack suddenly stops responding to your questions. | -1  contraindicated | 0  neither more or less indicated | 1  completely indicated |

| While having a walk-in the Saski Park, Lublin, you notice an elderly man gasping for breath, holding his left side, and having cold sweats. He complains of pain in the heart area and nausea. | | | | |
| --- | --- | --- | --- | --- |
| **If you were**  **thinking of:** | **And then:** | **This initial decision**  **becomes:** | | |
| Heart Attack (coronary event) based on exhibited symptoms. | The man has informed you that the pain is prickly and begins to radiate to the left shoulder. | -1  contraindicated | 0  neither more or less indicated | 1  completely indicated |
| Providing him with water to take his Aspirin, he took out of his pocket. | The man suddenly loses consciousness. | -1  contraindicated | 0  neither more or less indicated | 1  completely indicated |
| Sudden Cardiac Arrest, implementing Hands-Only- CPR. | A witness delivers AED from the nearby bank. | -1  contraindicated | 0  neither more or less indicated | 1  completely indicated |
| You are queuing at the grocery store. The 24-year-old man, a customer, standing in front of starts to stagger, supports himself on the counter, is pale, his hands are shaking, sweat appears on his forehead. He also utters incomprehensible words. After checking the ABC, you find that the man is breathing and has a heart rate, but although he is conscious, he has a problem verbalizing his responses. | | | | |
| **If you were**  **thinking of:** | **And then:** | **This initial decision**  **becomes:** | | |
| Hypoglycaemia | The man shows you: 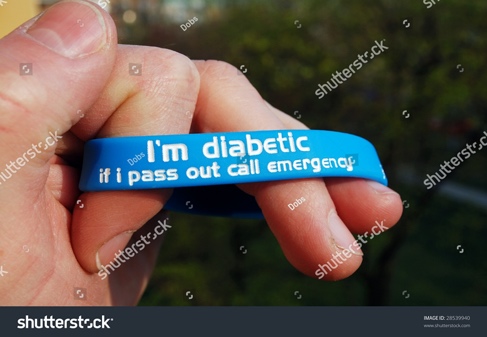 | -1  contraindicated | 0  neither more or less indicated | 1  completely indicated |
| Giving him a can of coke to drink as his glucometer shows a low value of blood glucose. | In the meantime, the man informs you that he has an ampoule of GlucaGen 1 mg HypoKit (glucagon). | -1  contraindicated | 0  neither more or less indicated | 1  completely indicated |
| After the man injects himself with the glucagon, you intend to take him to the closest ER. | In the meantime, you check the capillary refill, which is 5 seconds, and his breath is racing, more cold sweat appears on his forehead. | -1  contraindicated | 0  neither more or less indicated | 1  completely indicated |
